# Is health technology assessment ready for generative pretrained transformer large language models? Report of a fishbowl inquiry

**DOI:** 10.1017/S0266462324000382

**Published:** 2024-11-05

**Authors:** Clifford Goodman, Ellie Treloar

**Affiliations:** 1 Independent Consultant, Health Care Technology & Policy, Bethesda, MD, USA; 2 Discipline of Surgery, University of Adelaide, Adelaide SA, Australia

**Keywords:** technology assessment, biomedical, artificial intelligence, group processes

## Abstract

**Objectives:**

The Health Technology Assessment International (HTAi) 2023 Annual Meeting included a novel “fishbowl” session intended to 1) probe the role of HTA in the emergence of generative pretrained transformer (GPT) large language models (LLMs) into health care and 2) demonstrate the semistructured, interactive fishbowl process applied to an emerging “hot topic” by diverse international participants.

**Methods:**

The fishbowl process is a format for conducting medium-to-large group discussions. Participants are separated into an inner group and an outer group on the periphery. The inner group responds to a set of questions, whereas the outer group listens actively. During the session, participants voluntarily enter and leave the inner group. The questions for this fishbowl were: What are current and potential future applications of GPT LLMs in health care? How can HTA assess intended and unintended impacts of GPT LLM applications in health care? How might GPT be used to improve HTA methodology?

**Results:**

Participants offered approximately sixty responses across the three questions. Among the prominent themes were: improving operational efficiency, terminology and language, training and education, evidence synthesis, detecting and minimizing biases, stakeholder engagement, and recognizing and accounting for ethical, legal, and social implications.

**Conclusions:**

The interactive fishbowl format enabled the sharing of real-time input on how GPT LLMs and related disruptive technologies will influence what technologies will be assessed, how they will be assessed, and how they might be used to improve HTA. It offers novel perspectives from the HTA community and aligns with certain aspects of ongoing HTA and evidence framework development.

## Introduction

The scientific program of the Health Technology Assessment International (HTAi) Annual Meeting held 24–28 June 2023 in Adelaide, Australia, included a novel “fishbowl” session to gain diverse attendees’ input on the potential role of HTA in the emergence into health care of generative pretrained transformer (GPT) large language models (LLMs). The session, “Is HTA Ready for GPT/AI Large Language Models? A Participative Fishbowl Session” was the first time that HTAi used the fishbowl format at an annual meeting.

GPT LLMs, a set of artificial intelligence (AI) chatbots, accelerated into broad public awareness with the launch of the freely available ChatGPT by OpenAI on 30 November 2022. OpenAI stated that ChatGPT “enables users to refine and steer a conversation towards a desired length, format, style, level of detail, and language” (1).

The emergence of GPT LLMs such as ChatGPT was accompanied by current and projected applications, including creating human-like dialogue (e.g., in question-and-answer or point-counterpoint formats); creating literary, scientific, and artistic content; translating text into various languages; generating research syntheses; generate computer programming code; and more. Such applications were envisioned in virtually any industry. At the same time, concerns arose about the unintended uses and risks of GPT LLMs, including that they could, for example, generate and spread misinformation and disinformation, threaten privacy and security, substitute for certain forms of employment, and diminish human creativity (2–4).

Given their broad, potential, known, and unknown applications in health care, ChatGPT and other GPT LLMs merit the attention of the field of HTA. Doing so may better position and prepare HTA to anticipate and assess the unintended and intended consequences of these emerging, powerful, and likely far-reaching technologies.

The initiative for holding a fishbowl-type session on this topic during the HTAi 2023 Annual Meeting was taken by the HTAi Secretariat and planned in collaboration with Clifford Goodman, who also facilitated the fishbowl session. Ellie Treloar took detailed notes during the session, supporting much of the content that is summarized below. Rossella Di Bidino provided support on technical aspects of related AI subject matter.

## Fishbowl process

Various forms of structured and semistructured, interactive, group deliberation include such processes as the Delphi technique, nominal group technique, brainstorming, and various forms of consensus development. The “fishbowl” process is an interactive format for conducting medium-to-large group discussions. Fishbowls can be useful for sharing insights and other information from a variety of perspectives on emerging “hot topics.” These topics are addressed in a set of typically open-ended questions posed by a facilitator. Rather than having an unstructured discussion among a large group, participants are separated into an inner group of several (e.g., three-to-six) people seated in a circle or semicircle. On the periphery of the inner group is an outer group of perhaps no more than 30–50 participants. Supported by a designated facilitator, the inner group focuses on a series of a few or several discussion questions, whereas members among the outer group listen actively without interrupting and consider what they might want to offer to the discussion.

During the course of the fishbowl, participants voluntarily enter and leave the inner group. As such, inner group participants choose when to move to the outer group, typically after having offered one or more comments to the question at hand. Outer group participants may choose to enter the inner group by filling an open seat when they have comments to offer on that question. Outer group members may also choose to kindly tap the shoulder of an inner group member after the inner group member has commented on the question at hand, seeking to assume that person’s seat. In some versions of the fishbowl, participants are instructed to maintain one open seat, so that when an outer group participant comes to take that open seat, an inner group participant voluntarily moves to the outer group. Participants may enter and leave the inner group more than once during the course of a fishbowl (5–7).

The facilitator seeks to ensure that participants address the discussion question, all participants in the fishbowl have the opportunity to speak, and the rotation of the inner and outer groups proceeds. The facilitator may ask inner group participants to clarify their comments briefly to ensure that those comments are understood and address the present discussion question.

## Annual Meeting 2023 Fishbowl

As this was the initial fishbowl experience for most participants, the facilitator explained the format at the outset. For this seventy-five-minute fishbowl, six seats were designated for the inner group, plus a seventh seat for the facilitator. Approximately seventy people attended all or most of the session, of whom thirty-seven representing fifteen countries took part in the inner group. Participants were attendees of the HTAi 2023 Annual Meeting and were not pre-enrolled or otherwise designated prior to the fishbowl session. Apart from their self-selection indicating interest in this session, no assumptions were made regarding their levels of prior knowledge of AI, GPT LLMs, or related topics.

The process was guided in part by a short PowerPoint slide deck that introduced the purpose of the session (Table 1) and a set of interrelated definitions to provide a shared terminology and enable wider participation (Table 2), followed by three main questions for focused discussion.

**Table 1.** Why this fishbowl session?

A fundamental tenet of HTA is to examine the *intended* as well as *unintended* impacts – *beneficial or harmful* – of technology. Generative pretrained transformer (GPT) large language models (LLMs) (such as ChatGPT) have the potential to disrupt healthcare decision making and delivery. Today, we will: Identify current and potential future applications of GPT LLMs in health care Propose how HTA can assess (and adapt if needed) intended and unintended impacts of GPT LLM applications in health care Propose how GPT could be used to improve HTA methodology

**Table 2.** Terminology to support fishbowl discussion

A**rtificial intelligence (AI**) is a simulation of human intelligence processes by machines, especially computer systems, including perceiving, synthesizing, and inferring information. **Machine learning (ML)** is an AI method that teaches computers to learn from experience. Machine learning algorithms use computational methods to “learn” information directly from large volumes of data to classify data, find patterns, and make predictions. Three main types of ML are: Supervised: feed labeled training sets to the machine to help it learn (e.g., classify) Unsupervised: machine “looks for” patterns in data on its own Reinforcement: machine checks with data environment to confirm or correct its classifications, patterns, or predictions (learn from mistakes) **Artificial neural networks** are computing systems inspired by the biological neural networks in animal brains. They are typically groups of interconnected nodes, arranged into multiple layers, with signals passing from an input layer through intervening layers (possibly multiple times) to an output layer. **Deep learning** is a subset of machine learning that relies on multilayered artificial neural networks. Requires massive volumes of labeled data and high-performance computing Examples of applications: computer vision, speech recognition, speech translation, natural language processing (NLP), medical image analysis, drug design, bioinformatics, climate science, board games, self-driving cars, credit card fraud detection, satellite object detection Transformers (as in generative pretrained transformers [GPT]) are a type of deep learning model. **Natural language processing (NLP)** is an application of AI (deep learning in particular), computer science, and linguistics that is used in information retrieval, information extraction, and translation. Examples of applications: email filtering, online searches, document analysis, predictive text, social media monitoring, chatbots, language translation **Generative pretrained transformer (GPT)** models are artificial neural networks based on transformer architecture (a deep learning model), pretrained on large data sets of unlabeled text, and able to generate novel human-like content. **Large language models (LLMs)** are computer programs that can generate natural language text in response to given inputs LLMs have been trained through deep learning algorithms to recognize, generate, translate, and/or summarize massive quantities of textual data (articles, books, Web sites, etc.) Examples: GPT–3, GPT–4 (OpenAI), LLaMA (Meta), PaLM2 (Google)

*Source*: Authors’ synthesis of various definitions and descriptions.

The facilitator helped to ensure that participants rotated between the inner and outer groups throughout the session. Given that some participants were initially reticent about taking seats in the inner group, the facilitator called on several people to fully populate the initial inner group and occasionally signaled to outer group participants to suggest that they enter the inner group, while seeking to maintain a mix of national and regional representation throughout. Participants responded to each of the three main discussion questions with original points as well as reacting to others’ points, including clarifying, augmenting, or otherwise elaborating on those points.

## Fishbowl findings

The following are summaries of participants’ responses to each of the three main questions posed for this fishbowl. Fishbowl participants offered approximately sixty distinct responses across the three questions. Rather than verbatim listings of these responses, these summaries reflect categorization of the responses by subheadings for each question, consolidation of responses to reduce redundancies, and edits for clarity. Given the semistructured flow of the fishbowl, participants sometimes offered responses that were better suited to another of the three questions than the one that was presently being discussed; such responses are included under the appropriate questions’ response summaries below.

### Question 1: What are current and potential future applications of GPT LLMs in health care?

Responses to this question fell under six subheadings: patient care, patient self-care and companionship, clinician training and education, clinical trials, evidence and literature syntheses, and terminology and language. Given the rapid evolution and diffusion of GPT LLMs and varying levels of participants’ knowledge of them, the discussion made only limited distinctions between current and potential future applications.

#### Patient care

- Interact with patients to collect and consolidate their input, for example:
  - Improve the efficiency of generating electronic medical records (EMRs) and other reports
  - Improve patient centricity
- Provide talk therapy
  - Some people prefer not to speak with machines
- Improve various hospital operations and related tasks and follow-ups
- Search, screen, and analyze large amounts of online sources to discern patients’ and other users’ real health-related needs and concerns, including those that may not be expressed to or heard by clinicians
  - Include users’ online searches, social media content, and other communications to understand what information they seek, how they communicate and express themselves, and their related needs and concerns
- Generate improved (e.g., user-friendly) clinical communications with patients
- Translate or adapt clinicians’ responses and other communications to patients for more empathetic, kinder delivery
- Generate questions and discussion guides for, e.g., patient histories, interviews, and focus groups
- Provide tools that reduce the resource strain of routine communications and other administrative tasks for general practitioners and other clinicians

#### Patient self-care and companionship

- Provide human-like companionship for patients who feel lonely or isolated
- Provide communication tools to substitute for unnecessary or inappropriate use of clinician appointment time

#### Clinician training and education

- Generate training and education resources tailored for specific clinical and other professions and levels of experience and education
- Help train clinicians (e.g., with simulations) to better understand and communicate with patients, including how to ask better questions
  - Generate interactive patient models/avatars for training medical students and other healthcare professionals (e.g., taking health histories and conducting talk therapy)
- Training algorithms must be based on valid, diverse, and unbiased information sources

#### Clinical trials

- Develop interactive communication tools for patient identification and recruitment for clinical trials

#### Evidence and literature syntheses

- Improve the efficiency of systematic reviews, including improving the identification of potentially relevant studies, screening for inclusion, data extraction, and summarizing findings
- Transform relevant journal literature into clinical practice guidelines and other protocols using, for example, natural language processing
- Augment evidence from clinical research with real-world literature and big data sources for comparison to, and for purposes of updating, clinical practice guidelines
- Assemble dossiers on health technologies for submission to regulatory authorities to gain market approval or clearance
  - Review, evaluate, and generate summaries of such dossiers
- Manage GPT LLM algorithms/models and data sources to reduce generation of hallucinations (i.e., fabrications or other false information presented as facts) and other misinformation
- Evaluate GPT LLM summaries and other outputs for bias and misinformation

#### Terminology and language

- Standardize and otherwise improve healthcare lexicons and appropriate use of terminology
- Reduce language barriers
  - Translate communications into languages as needed, including rare ones for which there are few if any available human translators
  - Recognize GPT LLM and other AI limitations in achieving human-level understanding
- Convert heterogeneous patient-level and other non-professional input into clinician-level/professional language
- Generate plain-language (i.e., for nonexperts) and bespoke (e.g., to particular grade levels) documentation (e.g., patient instructions)

### Question 2: How can HTA assess (and adapt if needed) intended and unintended impacts of GPT LLM applications in health care?

Responses to this question fell under eight subheadings: HTA terminology; establishing human comparators; outcomes for assessing GPT LLMs; ethical, legal, and social impacts and implications; potential biases in source data and evidence; transparency; expertise and knowledge transfer for assessment; and relevant regulatory and legal requirements, as follows.

#### HTA terminology

- Define or standardize terminologies used in GPT LLMs in health care to clearly characterize them and formulate HTAs, for example, for:
  - Use in PICOTS (population, intervention, comparator, outcomes, time period, setting) frameworks

#### Human comparators

- Define or establish standards for human comparators for assessing the impacts of GPT LLM applications for performing tasks that are normally performed by humans

#### Outcomes and other end points

- Specify outcomes and other end points for assessing GPT LLMs, for example:
  - Reduced time to conduct certain tasks
  - Reduced clinician burden
  - Patient/user feedback on usefulness and acceptance of GPT LLM applications, for example:
    - Reduced loneliness and other emotional aspects
    - Using this feedback to improve the GPT LLM applications
  - GPT LLMs could generate or support subgroup analyses and reports for different population segments
  - Outcomes and other performance of GPT LLM applications may shift over time for those that are based on machine learning systems

#### Ethical, legal, and social impacts/implications

- GPT LLMs will raise ethical, legal, and social implications that call for updating HTA frameworks
- Patients may be unable to distinguish between interacting with a (human) clinician or a machine; not being informed of this may raise legal concerns
  - Applicable when a machine can pass the “Turing test” (i.e., exhibit intelligent behavior indistinguishable from that of a human)
- Given the emerging nature of these technologies, it may be difficult at this time to anticipate these implications

#### Potential biases in source data and evidence

- Carefully review the information and data sources on which the GPT LLMs (and other systems relying on machine learning) are based to identify potential incompleteness, inaccuracies, biases (e.g., skewed representation of patient groups, publication bias), and other aspects that may diminish their usefulness for their intended applications
  - Include the source data/information sets used to train, validate, and test a GPT LLM
  - Using the same data/information to train, validate, and test a GPT LLM may give a false indication of its performance
  - Confirmed and potential sources of incompleteness, inaccuracy, or bias should be identified, documented, and made transparent for the assessment process
- Need to assess algorithms or other models of the GPT LLMs for any errors or potential biases that may yield false (e.g., hallucinations), inaccurate, or otherwise potentially harmful outputs
- Recognize that GPT LLMs that use biased data/information, or that use biased (or otherwise faulty) logic, may propagate those biases in healthcare decision making

#### Transparency

- Make transparency in GPT LLM platforms, models, algorithms, and related AI applications a tenet of HTA
  - Describe why particular GPT LLMs or other AI tools were used
  - Ability to “take it apart” and understand how it works
  - Scrutinize underlying programming code where available and accessible
  - Transparency and verification are especially important for technologies with potential critical impact on safety and/or effectiveness
- Determine whether GPT LLMs are implemented as designed and indicated, including performance in real-world settings
- Determine whether GPT LLMs are accompanied by sufficient education and transparency for patients/users about how they work and their limitations

#### Expertise and knowledge transfer

- Involve experts in GPT LLMs and related aspects of AI in HTA processes
  - Ensure interactive collaboration and knowledge transfer among HTA personnel, experts, and other stakeholders

#### Regulatory requirements and status

- Gain familiarity with and access to expertise as needed regarding copyright, licensing, certification, privacy, security, marketing approval, and other legal and regulatory requirements of GPT LLMs being assessed

### Question 3: How might GPT be used to improve HTA methodology?

Responses to this question fell under four subheadings: improving the efficiency of HTA functions, advancing HTA frameworks and related research design, improving data analysis and reporting, and stakeholder engagement in HTA.

#### Efficiency of HTA functions

- Improve staff efficiency by off-loading certain labor-intensive functions or tasks to GPT LLMs to, for example:
  - Automate literature searches and systematic reviews
  - Draft systematic reviews and other narrative syntheses
  - Draft routine and bespoke (tailored) documentation for internal and external users
  - Function as editing and proofreading assistant
- Summarize available cost-effectiveness and other related economic studies on a particular topic and generate recommendations for appropriate modeling for further inquiry

#### HTA frameworks and research

- Generate draft PICOTS and other frameworks for conducting HTAs
- Identify and summarize HTA findings and recommendations from around the world
- Suggest research designs and models (e.g., of cost-effectiveness) for particular types of assessments
- Generate draft/potential research hypotheses from existing clinical trials and other research reports

#### Data analysis and reporting

- Generate programming code to conduct searches and extract data, for example, from EMRs
- Identify, analyze, and report on adverse events and other outcomes from EMRs and other large data sets
- Improve patient/population diversity in data and evidence capture and synthesis, including from nontraditional or difficult-to-access sources

#### Stakeholder engagement

- Expand and deepen stakeholder engagement with GPT LLM communications and resources, for example, educational resources, translations, plain-language summaries, and other knowledge transfer
- Collect and summarize patient experience and perspectives; generate simulated patient experiences and case studies

## Discussion

As GPT LLMs and other AI-based healthcare technologies emerge and diffuse into the healthcare sector, the field of HTA has begun to examine their potential mutual impacts. For example, a systematic review of the early literature on AI-based healthcare technologies identified and compiled challenges posed by such technologies to current frameworks of healthcare technology regulation and HTA (8). Another systematic review examined whether the types and quality of evidence generated from clinical trials and other studies of AI-based medical devices are sufficient to enable sufficiently comprehensive HTA, and called attention to how current HTA frameworks could be adapted to better assess these technologies (9). HTA organizations that are engaged in horizon scanning or otherwise anticipating the need to assess AI-based or -enabled healthcare technologies are acknowledging the need to adopt or develop criteria, evidence requirements, or modified frameworks accordingly (10–15). A high-level perspective suggests that HTA should recognize that adoption of AI-based systems will bring about broader health system transformations, well beyond the particular impacts of discrete technologies (16).

Though not focused on GPT LLMs in particular, several HTA organizations have recently developed and updated general evidence standards and related frameworks for evaluating digital health technologies, including those using AI (17–19). Also of potential relevance to HTA are recently developed international guidelines and tools intended to strengthen and assess the quality of research reports of AI-enabled technologies in such areas as medical imaging, diagnostic accuracy, predictive model bias assessment, and clinical trials reporting (20). Complementary to the types of work noted above, the results of this fishbowl process offer original HTA community input as well as alignment with certain aspects of ongoing HTA and evidence standards development.

The three main questions posed to the participants were distinct in that they addressed current and potential applications of GPT LLMs in health care, how HTA would assess these applications, and how GPT might be used to improve HTA methodology itself. Among the prominent and overlapping themes arising across the three main questions were improving operational efficiency, terminology and language, training and education, evidence synthesis, detecting and minimizing biases, stakeholder engagement, and recognizing and accounting for ethical, legal, and social implications.

Although internationally diverse, this group of participants was not intended to fully represent the international HTA community, as it was subject to self-selection arising from one’s ability to attend an international event, having an interest in the topic, and being available for the designated session time slot. Subject to the rotation of participants inherent in the fishbowl process, the flow of discussion was not consistently linear in that participants needed to wait for an opportunity to enter the inner group and provide their input. The ability to contribute to the discussion may have been limited for some participants unsure about their use of new terminology or whose first language was not English. The summaries reported here of participants’ responses to the fishbowl questions reflect their views expressed at the time of the HTAi 2023 Annual Meeting.

## Conclusion

The HTAi 2023 Annual Meeting presented an opportunity to engage an international group in taking an early, open-ended look at an emerging and likely disruptive set of technologies. The fishbowl format provided an interactive, face-to-face means of gaining real-time input from a diverse group of participants on how these technologies will influence what is assessed by HTA organizations, how they are assessed, and how HTA itself is conducted. This was a novel format for the HTAi Annual Meeting, designed to elicit and share original thinking on a focused set of issues subject to a set time constraint in the context of a busy international event. Participants were able to contribute to, absorb, and incorporate for further use foundational concepts of the role of HTA in GPT LLMs and related AI applications. For many participants, this fishbowl provided entry into global deliberations on these emerging issues. Furthermore, this demonstrates a model of a deliberative group process that they may choose to apply for other HTA-related purposes, and that HTAi might employ for future meetings and other functions.
